# Dengue virus co-infections with multiple serotypes do not result in a different clinical outcome compared to mono-infections

**DOI:** 10.1017/S0950268820000229

**Published:** 2020-06-29

**Authors:** U. T. N. Senaratne, K. Murugananthan, P. D. N. N. Sirisena, J. M. Carr, F. Noordeen

**Affiliations:** 1 Department of Microbiology, Faculty of Medicine, University of Peradeniya, Peradeniya, Sri Lanka; 2 Department of Multidisciplinary Sciences, Faculty of Allied Health Sciences, General Sir John Kotelawala Defence University, Werahera, Sri Lanka; 3 Department of Microbiology, Faculty of Medicine, University of Jaffna, Jaffna, Sri Lanka; 4 Microbiology and Infectious Diseases, College of Medicine and Public Health, Flinders University, Adelaide, Australia

**Keywords:** Dengue, DENV co-infections, RT-PCR, Sri Lanka

## Abstract

Circulation of multiple dengue virus (DENV) serotypes in a locale has resulted in individuals becoming infected with mixed serotypes. This research was undertaken to study the clinical presentation, presence of DENV serotypes and serological characteristics of DENV infected patients with co-infections from three Provinces of Sri Lanka where DENV-1 and -2 predominated during the study. A reverse transcription polymerase chain reaction was performed on 1249 patient samples and 301 were positive for DENV (24.1%). DENV-1 was the predominant serotype detected in 137 (45.51%) followed by DENV-2 in 65 (21.59%), DENV-3 in 59 (19.6%) and DENV-4 in 4 (1.32%) patients with mono-infections. Thirty-three patients (10.96%) had DENV co-infections with two or more serotypes. The highest number of co-infections was noted between DENV-1 and DENV-2 (57.57%) suggesting co-infection is driven by the frequency of the circulating serotypes. Platelet counts were significantly higher in DENV co-infected patients although clinical disease severity or white blood cell count, packed cell volume or viraemia were not significantly different in the co-infected compared to the mono-infected patients. Thus co-infection with multiple DENV serotypes does occur but with the exception of improved platelet counts in co-infected patients, there is no evidence that clinical or laboratory measures of disease are altered.

## Introduction

Dengue fever (DF) is a mosquito borne infection caused by four antigenically distinct virus serotypes, namely DENV-1 to 4. Sri Lanka lies in the dengue endemic region where dengue virus (DENV) infection and numerous cases of DF and dengue haemorrhagic fever (DHF) have been reported since the 1960s [1,2]. Before 1989, all four DENV serotypes were known to circulate in Sri Lanka [3]. However, over time, specific serotypes have emerged to cause outbreaks. Previous studies have also indicated strain variations with time that have been linked to the change in disease severity, for example, DENV-3 has shown strain differences following the emergence of DHF [4]. Studies on circulating DENV serotypes in Sri Lanka, including our own analysis from 2012 indicate DENV-1 to be the most prevalent serotype in the Western [2] and Central Provinces [5]. Further, sequence analysis of the DENV-1 strains detected in 2012 demonstrated a close relationship to the DENV-1 strains described earlier in 2009–2010, indicating little strain variation in recent years [5]. Co-incident with this 2012 DENV-1 analysis, the presence of all four DENV serotypes [5] demonstrated in the Central Province of Sri Lanka, indicates hyper-endemicity. This might be due to the spread and expansion of the mosquito vector resulting from rapid urbanisation and development in the area [1,2]. DENV hyper-endemicity has also been reported from other countries that have been undergoing rapid urbanisation and development, such as in New Caledonia, Thailand, China, Somalia and Brazil [6–10]. The circulation of multiple DENV serotypes in the same locale has caused co-infections with different DENV serotypes in patients in subsequent or simultaneous infections and this is shown to be associated with increased risk for severe dengue. Co-infection may occur during outbreaks in countries where multiple DENV serotypes co-circulate allowing the same mosquito to feed on more than one DENV infected individual, potentially acquiring and transmitting multiple DENV serotypes at subsequent blood meals [11,12]. The first report of co-infection or super infection with multiple DENV serotypes in a single patient was from Puerto Rico in 1982 [11] and several cases have been reported thereafter [7,12–15]. However, the presence of two or more serotypes in one patient has not been previously reported from Sri Lanka, although multiple DENV serotypes have been known to co-circulate since 1960s.

Numerous publications are available on the clinical presentations, relationship between the DENV viral load or virus types and the disease severity in DENV mono-infection [16,17]. In contrast, there is less literature available describing changes in clinical parameters in co-infected DENV patients. Hence, the objective of the current study was to define the presence of mixed infections with multiple DENV serotypes in patients from three Provinces across Sri Lanka and to analyse the haematological parameters, differences in serotypes and viraemia among mono- and co-infected patients. We demonstrate both similarities and unique aspects of clinical and laboratory profiles in patients with DENV mono-infections when compared with co-infections. These results facilitate the understanding of the impact of mixed infections with 2 or more DENV serotypes on dengue and add to our understanding of the potential for diversity of DENV infections in a patient in endemic countries.

## Methods

This is a cross sectional study involving patients' clinical, haematological data and samples collected from three Provinces of Sri Lanka, representing the Central Province (Group 1), Western Province (Group 2) and the Northern Province (Group 3). The group 1 samples were collected from July 2011 to February 2012, group 2 samples were collected from July 2011 to June 2012 and group 3 samples were collected during two outbreaks in the Northern Province 2009–2010 and 2011–2014.

The clinical and haematological data were obtained with informed written consent (2011/EC/08, 2011/EC/13 and 2011/EC/49) and entered into a detailed questionnaire from 1249 clinically suspected DF/DHF patients presenting with fever ⩽5 days (onset of fever was considered as day 1) according to the DF/DHF classification guidelines [18]. Blood was also collected from patients and tested as below.

### DENV serology

Both anti-DENV IgM and IgG detection were performed by capture ELISAs following manufacturer's instructions (Standard Diagnostics, Korea). When only anti-DENV IgM was positive it was considered as a primary DENV infection and when either IgG alone or IgG with IgM showed positivity it was considered as a secondary DENV infection.

### RT-PCR of patients' sera

**Fig. 1.**
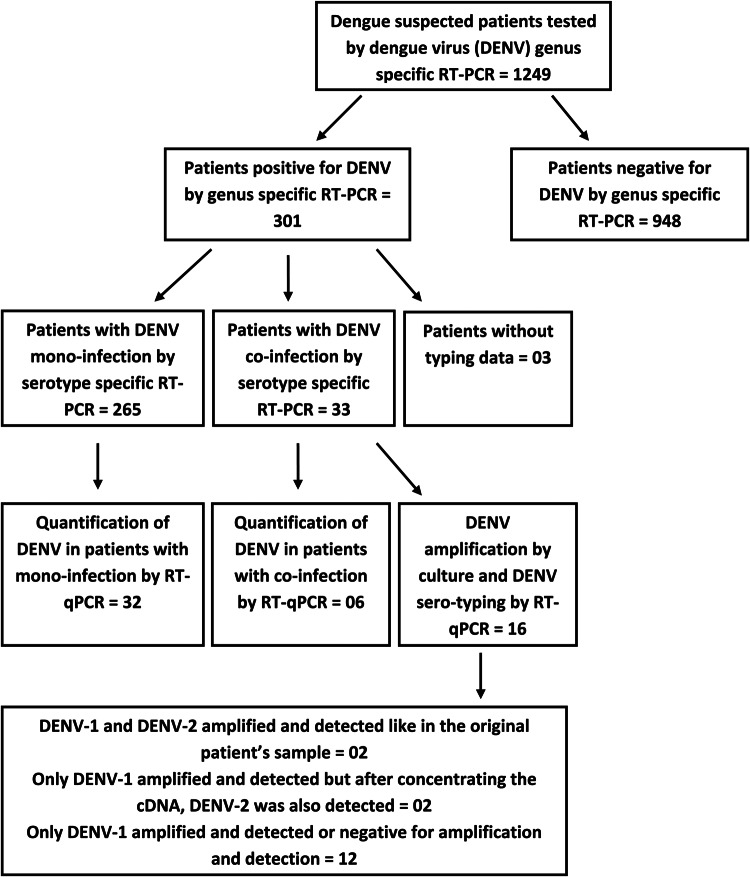
A flow diagram illustrating the detection of DENV infection followed by the detection of DENV mono and co-infections in the study sample; testing subsets of patients' samples using the RT-qPCR for DENV quantification and amplification by culture and DENV sero-typing.

The viral RNA was extracted from patient sera (*n* = 1249) using a standard RNA extraction system (Qiagen, Hilden, Germany) (Fig. 1). Two types of DENV reverse transcription polymerase chain reaction (RT-PCR) were performed using NS3 consensus primers DV1/DV3 [19] and D1/D2 capsid (C) primers [20], which detect DENV serotypes 1–4 and we have previously defined to successfully amplify Sri Lankan DENV strains [5]. The DENV genus positive samples [19] were serotyped across the C region of the genome using previously published primers [20]. DENV serotypes were identified by the RT-PCR on RNA extracted from patient sera and also from *in vitro* amplified virus, where possible. The RT-PCR was performed using a Swift Max thermocycler (Esco Healthcare, Singapore) followed by the detection of the PCR products by agarose gel electrophoresis. A single step RT-PCR was performed with specific primer pairs for each DENV serotype separately. Hence, for the dual or triple infections, two or three PCR reactions, respectively, were performed using the primers for the respective DENV types in separate tubes.

### Quantification of virus load in the sera of patients with DENV infections

The virus load was quantitated for 38 DENV RNA extracts from patients' sera with the C region of the genome using previously published primers for DENV [20]. Reactions used the GoTaq 1-step RT-qPCR system, as per manufacturer's instructions (Promega, USA; Cat #A6020) and were performed in a Qiagen rotor gene PCR machine (Qiagen, Hilden, Germany) with SyBr green (Promega USA) detection and melt curve analysis of PCR products. Cycling conditions were used as follows: reverse transcription at 42 °C, 15 min, initial PCR activation at 95 °C, 10 min, 40 cycles of denaturation at 95 °C, 30 s, annealing at 55 °C, 30 s and 60 °C, 30 s (acquiring on green channel), extension at 72 °C, 1 min with a final extension at 72 °C, 10 min. Threshold cycles (CT) values were calculated and melt curve analysis was performed by ramping from 60 °C to 95 °C with 0.1 °C/10 s/cycle. Standard curves were obtained with titrated DENV-4 supernatants serially diluted from 1.9 × 10^6^ to 1.9 × 10^1^ PFU/ml. The *T*_m_ of each specific PCR product was analysed using Corbett Life Science Qiagen software.

### Amplification of virus from co-infected patient sera *in vitro*

Due to the limited availability of serum, virus was amplified from only 16 patient's sera by inoculating the serum sample onto C6/36 (*Aedes albopictus*) cells in tissue culture flasks for 3–7 days at room temperature. Supernatants were harvested, clarified, supplemented with 15% FBS and stored in aliquots at −80 °C. The 1st passage (P1) stocks were used as a source of RNA for extraction and serotyping as described above.

## Results

### DENV co-infections with different serotypes in dengue patients from Sri Lanka

The RT-PCR was performed on 1249 clinically suspected dengue patients and 301 were positive for DENV. These 301 patients' samples were subjected to DENV serotyping by the RT-PCR (Fig. 1). Results demonstrated a majority of DENV-1 infections with comparable levels of DENV-2 and DENV-3 and a few DENV-4 mono-infections (Table 1). Of the 301 PCR positive samples, three failed amplification and thus could not be serotyped. Repeated amplifications were not performed due to limited sample availability. Thirty-three of the 301 (10.96%) samples demonstrated the presence of two or more DENV serotypes (Table 1). Of the study population, five co-infected patients were from the Central Province (Group 1), 11 co-infected patients were from the Western Province (Group 2) and 17 co-infected patients were from the Northern Province (Group 3). Of the 33 patients in whom multiple DENV serotypes were detected, the DENV-1 and DENV-2 serotype combination was most commonly found, followed by the DENV-2 and DENV-3 co-infection and DENV-1 and DENV-3 co-infection. DENV-1 and DENV-4 or DENV-3 and DENV-4 co-infections and a triple co-infection of DENV-1, DENV-3 and DENV-4 were detected in single cases only patient (Table 1).

**Table 1.** Frequency of DENV serotype detection by the RT-PCR in patients' sera (*n* = 301).

DENV serotype/serotype combination	Frequency of DENV infections (*n* = 301) (*n*; %)	Frequency of DENV co-infection (*n* = 33) (%)
DENV-1	137; 45.5	–
DENV-2	65; 21.6	–
DENV-3	59; 19.6	–
DENV-4	4; 1.3	–
Number of mono-infections	265; 88	
DENV-1 and DENV-2	19; 6.3	57.6
DENV-1 and DENV-3	4; 1.3	12.1
DENV-1 and DENV-4	1; 0.3	3
DENV-2 and DENV-3	7; 2.3	21.2
DENV-3 and DENV-4	1; 0.3	3
DENV-1, DENV-3 and DENV-4	1; 0.3	3
Number of co-infections	33; 11	
Serotyping results not available	3; 0.9	

### DENV co-infected patients did not show any difference in infection outcome (DF or DHF) compared with mono-infected patients

The association between DENV co-infection with clinical assessment was analysed. Disease severity did not differ significantly (Fisher's exact test *P* = 0.95) in the mono-infected and co-infected population with 188/268 (70.1%) DF and 80/268 (29.9%) DHF patients infected with a single and 23/33 (69.7%) DF and 10/33 (30.3%) DHF patients infected with more than one DENV serotype. Based on the anti-DENV IgM and IgG data, the frequency of primary or secondary DENV was not different between mono-infected and co-infected patients. The predominance of DF/DHF was similar between the 2 subsamples (primary and secondary). In comparison, for the mono-infected population, the majority of primary and secondary infections were DF (Table 2). The distribution of the main two serotypes seen in co-infection (DENV-1/2) was comparable between DF and DHF (Table 2). Similarly, the numbers of DENV-1/2 co-infections were comparable between primary and secondary infections (Table 2). The numbers of patients with other DENV serotype co-infection combinations were too small to make any reliable conclusions.

**Table 2.** The association between DENV serotype combinations and disease severity (DF/DHF) in co-infections with respect to their anti-DENV IgM/IgG status

Final diagnosis	DENV serotype combination	Anti-DENV IgM/IgG status (*n*; %)		Total (*n*; %)
		Primary	Secondary	
DF (*n* = 23)	DENV-1 + DENV-2	5; 15.2	7; 21.2	12; 36.4
	DENV-1 + DENV-3	0	3; 9.1	3; 9.1
	DENV-2 + DENV-3	5; 15.2	0	5; 15.2
	DENV-1 + DENV-4	1; 3	0	1; 3
	DENV-3 + DENV-4	1; 3	0	1; 3
	DENV-1 + DENV-3 + DENV-4	0	1; 3	1; 3
DHF (*n* = 10)	DENV-1 + DENV-2	2; 6.1	5; 15	7; 21.2
	DENV-1 + DENV-3	0	1; 3	1; 3
	DENV-2 + DENV-3	2; 6.1	0	2; 6.1
Total		16; 48.5	17; 51.5	33; 100

### Haematogocial and virological parameters in DENV co-infected patients

**Fig. 2.**
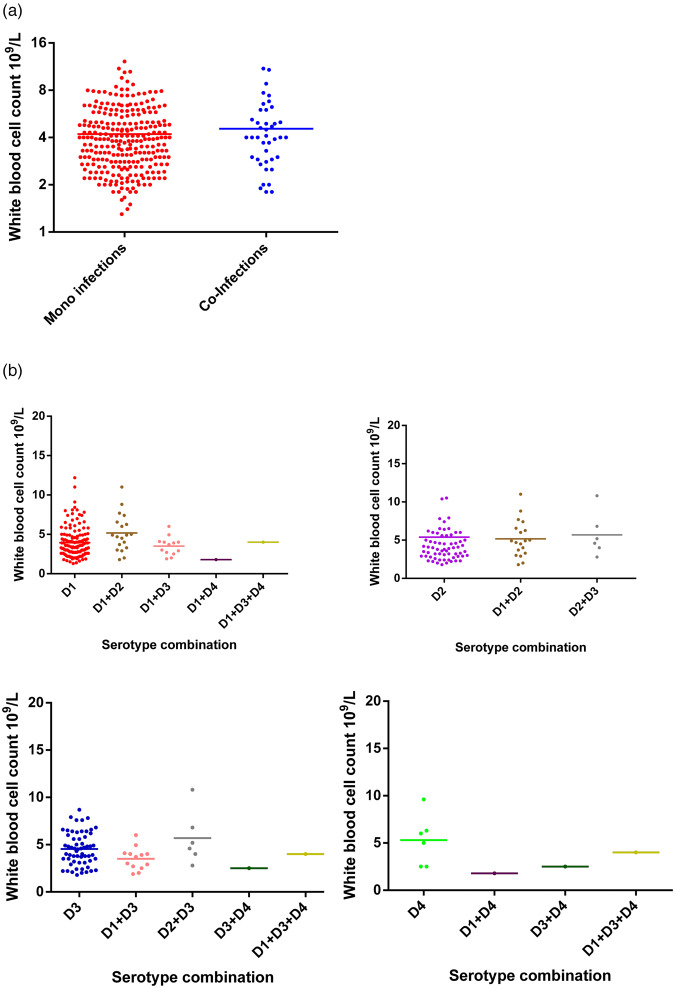
Comparison of WBC counts between DENV mono- and co-infections analysed by the Mann–Whitney test – DENV-1 (*n* = 137); DENV-2 (*n* = 65); DENV-3 (*n* = 59); DENV-4 (*n* = 6); DENV-1 + DENV-2 (*n* = 11); DENV-1 + DENV-3 (*n* = 13); DENV-1 + DENV-4 (*n* = 1); DENV-2 + DENV-3 (*n* = 6); DENV-3 + DENV-4 (*n* = 1); DENV-1 + DENV-3 + DENV-4 (*n* = 1). DENV is given as D in the figure and that D1 = DENV-1, D2 = DENV-2, D3 = DENV-3 and D4 = DENV-4.

Haematological data on white blood cell (WBC) count, platelet count and packed cell volume (PCV) was compared between mono- and co-infected DENV patients and mean ± s.d. are summarised in Table 3. Specifically, the WBC count did not differ between mono and co-infected patients (Fig. 2). Further dissection of these two groups based on serotype similarly showed no difference in WBC count between mono-infected DENV 1–4 or between the respective mono-infected serotype and co-infected serotype (Fig. 2). Although numbers were small, the three co-infections with DENV-4 tended to show lower WBC counts compared to mono-infection with DENV-4.

**Table 3.** Detection of DENV serotypes from the sera of patients and culture by the RT-PCR

Sample no	DENV serotypes detected	
	RT-PCR from serum	RT-PCR from culture
1	DENV-1 + DENV-4	PCR negative
2	DENV-4 + DENV-3	PCR negative
3	DENV-1 + DENV-3	PCR negative
4	DENV-1 + DENV-2	DENV-1 + DENV-2
5	DENV-1 + DENV-3 + DENV-4	PCR negative
6	DENV-1 + DENV-3	PCR negative
7	DENV-1 + DENV-2	DENV-1 + DENV-2
8	DENV-1 + DENV-2	DENV-1
9	DENV-1 + DENV-2	DENV-1
10	DENV-1 + DENV-2	DENV-1
11	DENV-1 + DENV-3	PCR negative
12	DENV-1 + DENV-2	DENV-1
13	DENV-1 + DENV-2	DENV-1
14	DENV-1 + DENV-2	DENV-1
15	DENV-1 + DENV-2	DENV-1
16	DENV-1 + DENV-2	DENV-1

**Fig. 3.**
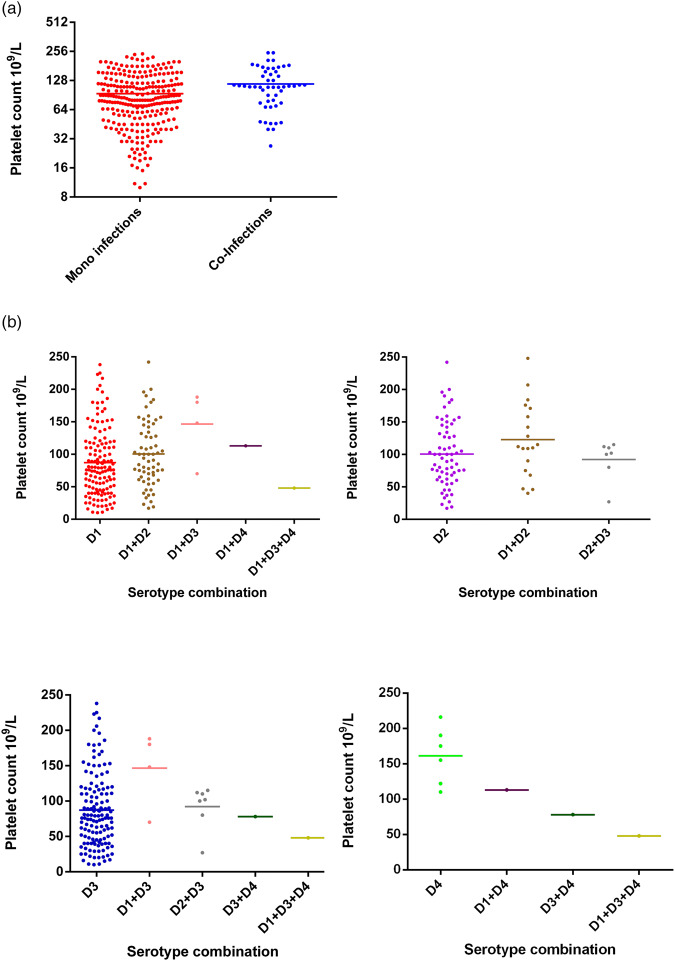
Comparison of platelet counts between DENV mono- and co-infections analysed by the Mann–Whitney test – DENV-1 (*n* = 137); DENV-2 (*n* = 65); DENV-3 (*n* = 59); DENV-4 (*n* = 6); DENV-1 + DENV-2 (*n* = 11); DENV-1 + DENV-3 (*n* = 13); DENV-1 + DENV-4 (*n* = 1); DENV-2 + DENV-3 (*n* = 6); DENV-3 + DENV-4 (*n* = 1); DENV-1 + DENV-3 + DENV-4 (*n* = 1); statistically significant*. DENV is given as D in the figure and that D1 = DENV-1, D2 = DENV-2, D3 = DENV-3 and D4 = DENV-4.

Platelet counts showed a significant increase in co-infected compared to mono-infected patients (Fig. 3; *P* = 0.002). Again, groups were stratified based on serotype (Fig. 3). Overall, co-infection with DENV 1/2, DENV 1/3 or DENV 1/4 tended to have higher platelet counts than DENV-1 alone, although this was not significantly different (Fisher's exact test *P* = 0.2747). In contrast, platelet counts for DENV-2 mono-infections were comparable to DENV-2 containing co-infections. The numbers of co-infections for DENV-3 and DENV-4 with types aside from DENV-1, however, were too small to make reliable conclusions.

**Fig. 4.**
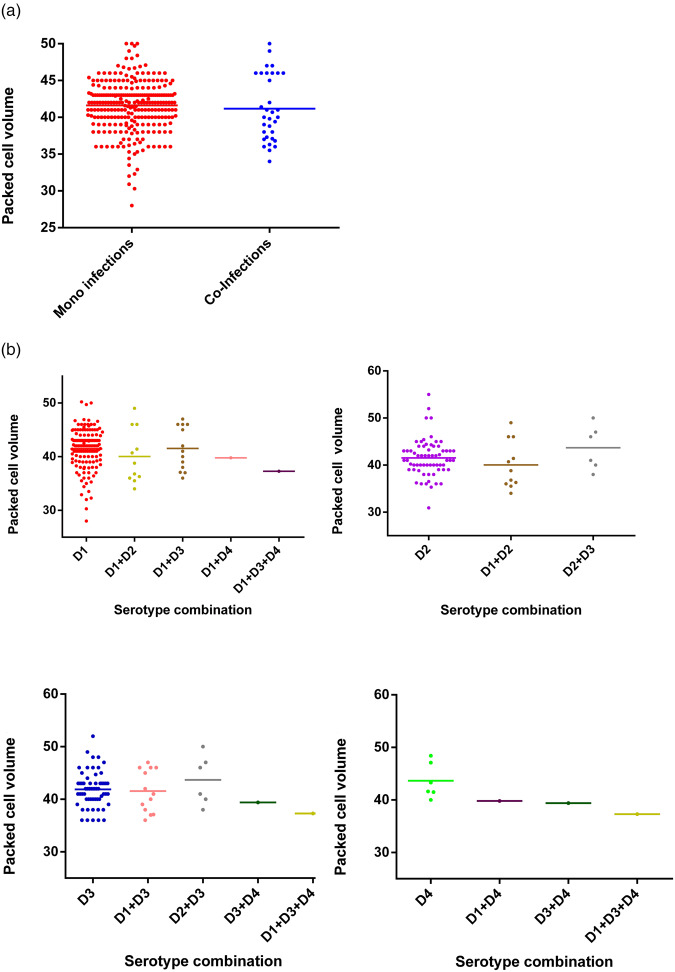
Comparison of PCV between DENV mono- and co-infections analysed by the Mann–Whitney test – DENV-1 (*n* = 137); DENV-2 (*n* = 65); DENV-3 (*n* = 59); DENV-4 (*n* = 6); DENV-1 + DENV-2 (*n* = 11); DENV-1 + DENV-3 (*n* = 13); DENV-1 + DENV-4 (*n* = 1); DENV-2 + DENV-3 (*n* = 6); DENV-3 + DENV-4 (*n* = 1); DENV-1 + DENV-3 + DENV-4 (*n* = 1). DENV is given as D in the figure and that D1 = DENV-1, D2 = DENV-2, D3 = DENV-3 and D4 = DENV-4.

Changes in PCV between mono-infections and co-infections were not statistically significant (Fisher's exact test; *P* = 0.4521), either considered as a whole group (Fig. 4) or further stratified by serotype (Fig. 4). Co-infections of DENV-4 with other serotypes showed reduction in PCV values even though the numbers of samples were too small to make statistically valid conclusions.

**Fig. 5.**
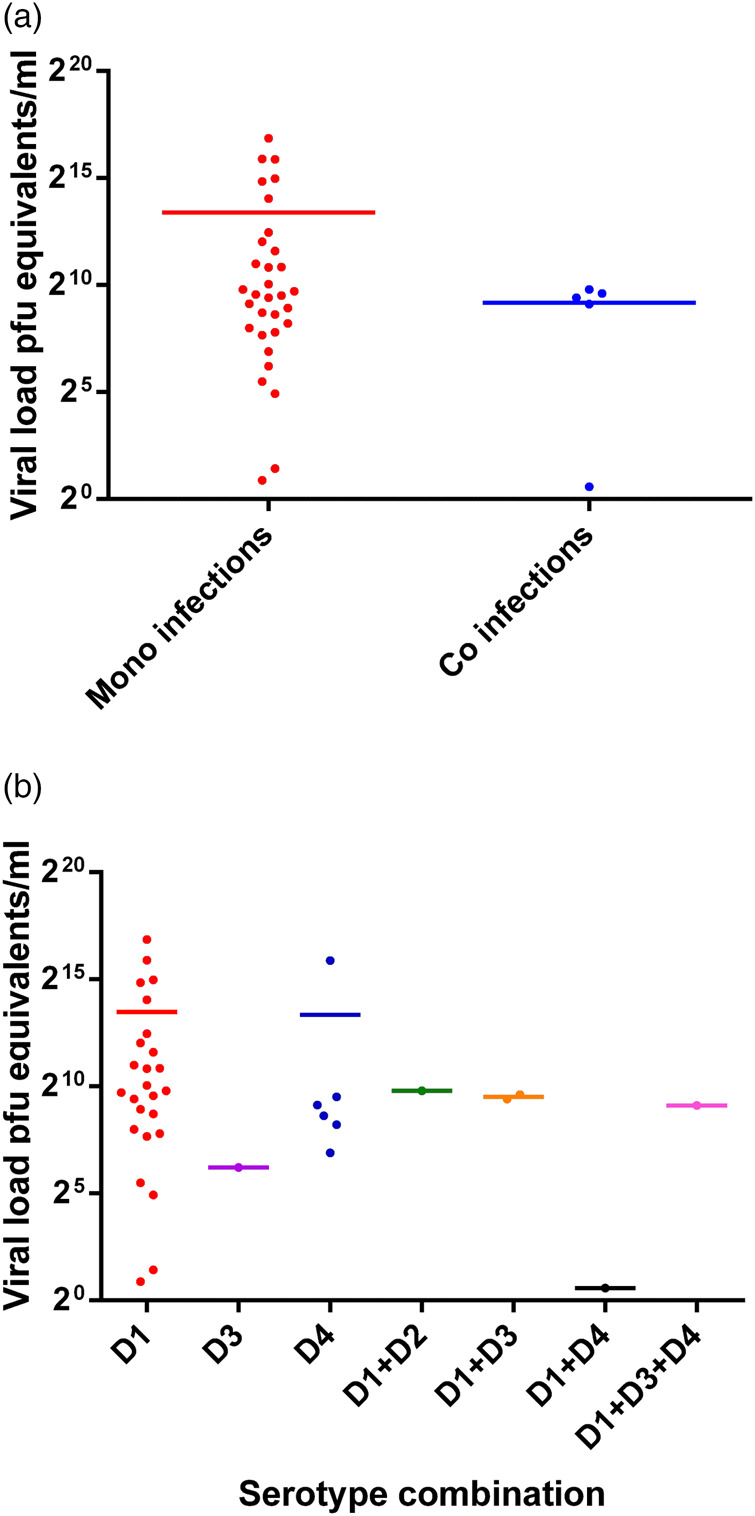
Comparison of the viral load between mono- and co-infections with different DENV serotypes (38 patients' samples were subjected to the quantitative RT-PCR using the capsid gene primers) [20]. DENV is given as D in the figure and that D1 = DENV-1, D2 = DENV-2, D3 = DENV-3 and D4 = DENV-4.

The viral load was quantitated in a subset of 38 patients (due to the limited availability of serum after the initial RT-PCR) and compared between mono (*n* = 32) and co-infected patients (*n* = 6). No significant difference (*P* = 0.3809) was observed in the group as a whole (Fig. 5) or following separation of results by DENV serotype (Figs 1 and 5). Although the single DENV1/4 co-infection analysed had a lower viral load, numbers in each group were low and thus reliable conclusions could not be drawn (Figs 1 and 5).

### Results of amplification of virus from co-infected patients' sera *in vitro*

Culture of patients' samples was performed on those identified as co-infections and the amplified virus in the culture supernatant was serotyped. Of the 16 available samples, 2 DENV-1 and DENV-2 co-infections amplified by serotypes, while the remaining samples either failed to amplify (PCR negative) or amplified DENV-1 serotype only (Table 3).

Two samples where both DENV-1 and DENV-2 were present in the initial patient material by the RT-PCR, the P1 stocks showed positivity for DENV-1 only but after concentrating the cDNA, DENV-2 was also detected (Fig. 1).

## Discussion

The presence of multiple DENV types in a single patient is possible when two or more serotypes circulate in the same locale. Patients may become infected with dual infections due to the feeding behaviour of *Aedes aegypti,* which feeds multiple times during a single gonotrophic cycle [21]. The ability of a single mosquito to transmit multiple serotypes at the same time could cause co-infections in a single patient [22]. Alternatively, a co-infection may result from ‘super-infection’ when bitten by a mosquito carrying one DENV serotype, closely followed by a bite from another mosquito carrying a second serotype.

The study herein started with over one thousand suspected dengue cases based on clinical diagnosis and serological analysis with fever for less than 5 days. Of these, the virus was detected by the RT-PCR in 23.3% (301 patients). In these 301, more than one DENV serotype was detected in 33 (10.9%). Several countries have previously reported concurrent DENV infections with different serotypes in the same patient at various percentages, 3% to 43% [6–9,14,21,23–25]. The vast difference in co-infection percentages reflects the dominance of different DENV serotypes in different countries and the capacity of the vector in transmitting DENV co-infections in these settings.

This is one of the first reports of the presence of multiple DENV serotypes in patients from three geographically distinct provinces from Sri Lanka. In our study population, the majority of co-infections showed DENV-1 and DENV-2 combination (Table 1) (*n* = 19). This may be due to the higher prevalence of mono-infections with DENV-1 followed by DENV-2 in the study sample. Thus, there is a strong trend towards the most predominant circulating DENV serotype to be present in more co-infections [14,23–27]. This suggests that co-infection with multiple DENV serotypes is most likely due to mathematical chance of infection, rather than a biological selection for a particular serotype.

Many co-infected patients' sera failed to grow DENV in culture (Fig. 1). In two samples where both DENV-1 and DENV-2 were present in the initial patient material by the RT-PCR, the P1 stocks had DENV-1 only. However, after concentrating the cDNA, DENV-2 was also detected. This culture bias for serotype growth may be due to the differences in the viral load of each serotype in the initial inoculum or competition in culture due to the relative biological fitness of the different serotypes. Similar discrepancies in culturing more than one serotype from co-infected patients' samples have been demonstrated [8]. However, a previous study points out that the virus antigen detection by an immunofluorescence assay is more sensitive over the RT-PCR and the decrease in the inoculum size together with prolonged incubation in culture allows better detection of virus [21]. Collectively, these factors may contribute to the difficulty in detecting the multiple serotypes in culture. Thus the applicability of culturing sera to identify multiple serotypes in the same sera is not reliable and needs exploration.

The impact of DENV co-infections on disease needs future studies. Dhanoa *et al*. [27] suggest enhanced disease severity in DENV co-infection. In contrast, in our study the co-infections consisted of 70% DF patients and 30% of DHF patients and co-infection did not affect the disease severity (Table 2). Platelet counts – a measure of disease severity, was higher in co-infected compared with mono-infected patients. This may be due to ‘super-infection exclusion with a homologous virus’ where the disease severity decreases when homologous infections coexist. This has not yet been demonstrated in humans but has been shown in mosquito cells [28]. It has been proposed that the reduced disease severity seen in viral co-infections might be due to the presence of defective viral particles [29].

The association of WBC, platelets and PCV counts with the disease severity during different DENV mono-infections has been described in Sri Lanka [5] and here we extend this with interesting changes in WBC (Fig. 2) and platelet counts (Fig. 3) and PCV (Fig. 4) in DENV co-infections compared with mono-infections (Supplementary Table S1). When DENV-1, DENV-2 and DENV-3 caused mixed infections, the WBC counts were always intermediate to the WBC counts of the respective mono-infection. However, when either of the above mentioned serotypes caused a mixed infection with DENV-4, the WBC counts were reduced compared to the respective mono-infections. The correlation of the presence of multiple infections with DENV-4 could not be statistically analysed due to the low number of samples with DENV-4 co-infections. Platelet counts in co-infections with DENV-1 and DENV-2 and also DENV-1 and DENV-3 were increased in comparison with mono-infections. This correlates with our suggestion of reduced disease severity in DENV co-infections. In contrast, the platelet counts for a co-infection with DENV-4 were either in-between the respective mono-infections or lower than the respective mono-infections. This association of DENV-4 with severe disease when combined together with other serotypes has been reported [15]. In our study sample, all co-infections with DENV-4 have shown DF indicating there may be differences in the behaviours of each serotype that might depend on the serotype combinations and DENV-4 strain variations. Co-infections of DENV-4 with other serotypes showed a reduction in PCV values even though the number of samples was small to make valid conclusions. A recent study in Malaysia found no association between the rise in PCV in co-infections [27].

Anti-DENV IgM and IgG results did not show a significant association between the antibody status of the patient and the type of the infection (mono- *vs.* co-infection) (*P* = 0.5823). This suggests that prior DENV infection does not reduce the chance of acquiring a co-infection and this further supports mathematical chance of exposure as the determinant of co-infection. However, no other literature is available to clarify or expand on these findings.

An elevated viral load has been associated with disease severity and secondary DENV infections in some studies [30,31] but not all [17]. The consensus seems to indicate more severe disease with a higher viral load. In this study, the viral load was determined in a subset of samples (*n* = 38) but showed no statistical difference between the mono- and co-infected patients (*P* = 0.3809). The mono-infected subset consisted of 30 DF and 4 DHF/DSS patients while all the co-infected patients had DF only and thus our study does not support more severe disease with DENV co-infections. In addition to the laboratory haematological measures, DENV co-infections may affect other parameters that can influence disease severity, such as differences in induction of vasoactive cytokines and this remains to be specifically assessed.

Aside from the impact of co-infections on outcomes for individual patients in terms of disease severity, high numbers of patients with co-infections with multiple serotypes may have a significant long-term impact on the future evolution of DENV. Sequencing of viral populations within a patient has demonstrated diversity of DENV sequences that in some studies is associated with more severe disease [32] but not in others [33]. Phylogenetically distinct DENV-1 recombinant strains have been documented within the same patient, suggesting a ‘mixed’ infection within the same serotype [33,34]. Theoretically, inter-serotype recombination could occur but evidence for this is lacking [35]. Perhaps with the changing epidemiology of DENV, with an increasing number of countries becoming hyper-endemic with multiple DENV types co-circulating, we may see this change in the future. Additionally, while this study was powered to assess associations with DENV-1/DENV-2 co-infections, larger numbers of DENV-3 and DENV-4 co-infected patients should be assessed to gain more sound conclusions on DENV-3 and DENV-4 co-infections and their associations with disease severity.

In conclusion, this is the first report of co-infections in a large sample of patients in Sri Lanka. Even though the proportion of mixed infections for some serotypes was small, this study sheds some light on the differences and similarities between mono and co-infections. Further studies are necessary with a higher number of co-infections, particularly for DENV-4 for a valid comparison between each mixed serotype combination. Disease associations were unchanged – platelet and WBC counts, PCV and viraemia supported the potential for less severe disease in co-infections. The impact of the presence of DENV co-infections on disease outcomes in individual patients is complex and adding to this complexity is the potential for DENV serotype co-infections to contribute to genetic diversity of DENV. Thus knowledge of DENV co-infections may help us understand the expanding global dengue epidemic.

## Supplementary material

For supplementary material accompanying this paper visit https://doi.org/10.1017/S0950268820000229. click here to view supplementary material
