# Supplementary material for: Dengue virus co-infections with multiple serotypes do not result in a different clinical outcome compared to mono-infections
Source: Epidemiol Infect. 2020 Jun 29;148:e119. doi: 10.1017/S0950268820000229 (PMC7325333; doi:10.1017/S0950268820000229)
Supplement: Supplementary file 1 [file S0950268820000229sup001.doc]

**Supplementary 1.** Haematological parameters in patients with DENV mono- and co-infections.

| **DENV serotype / DENV serotype combinations** | **Mean WBC**  **(± SD) x 10^9^ /L** | **Mean platelet**  **(± SD) x 10^9^/L** | **Mean PCV**  **(± SD) x 10^9^ /L** |
| --- | --- | --- | --- |
| **DENV-1** | 3.95 (± 3.95) | 87.18 (± 52.18) | 41.46 (± 3.75) |
| **DENV-2** | 5.40 (± 5.40) | 100.5 (± 50.22) | 41.54 (± 3.97) |
| **DENV-3** | 4.54 (± 4.54) | 93.17 (± 49.13) | 41.89 (± 3.38) |
| **DENV-4** | 5.32 (± 5.32) | 161.3 (± 40.54) | 43.65 (± 3.37) |
| **DENV-1 + DENV-2** | 5.18 (± 5.18) | 122.9 (± 57.20) | 40.05 (± 5.03) |
| **DENV-1 + DENV-3** | 3.51 (± 3.51) | 146.5 (± 53.85) | 41.55 (± 4.02) |
| **DENV-1 + DENV-4** | 1.80 (± 0) | 113.0 (± 0) | 39.80 (± 0) |
| **DENV-2 + DENV-3** | 5.70 (± 2.83) | 92.29 (± 31.05) | 43.67 (± 4.676) |
| **DENV-3 + DENV-4** | 2.50 (± 0) | 78.00 (± 0) | 39.40 (± 0) |
| **DENV-1 + DENV-3 + DENV-4** | 4.00 (± 0) | 48.00 (± 0) | 37.30 (± 0) |
